# Regulatory plasticity balances photosynthetic electron flow with enhanced pH-dependent cytochrome *b*_6_*f* control in Arabidopsis

**DOI:** 10.1093/plphys/kiag391

**Published:** 2026-06-19

**Authors:** Ryouhei Kobayashi, Zenpei Shimatani, Keiji Nishida, Toshiharu Shikanai

**Affiliations:** Department of Botany, Graduate School of Science, Kyoto University, Kyoto, Japan; Graduate School of Science, Technology and Innovation, Kobe University, Kobe, Hyogo, Japan; Graduate School of Science, Technology and Innovation, Kobe University, Kobe, Hyogo, Japan; Engineering Biology Research Center, Kobe University, Kobe, Hyogo, Japan; Department of Botany, Graduate School of Science, Kyoto University, Kyoto, Japan

## Abstract

To avoid photodamage of photosystem I under fluctuating light, plants have evolved multiple photoprotective mechanisms. One key mechanism is photosynthetic control, in which acidification of the thylakoid lumen downregulates electron transport through the cytochrome *b*_6_*f* complex, thereby preventing overreduction of photosystem I. The Arabidopsis *proton gradient regulation 5* (*pgr5*) mutant, which is defective in cyclic electron transport around photosystem I, fails to induce photosynthetic control and consequently suffers severe photosystem I photodamage under fluctuating light. Previously, we showed that introduction of the *pgr1* mutation, which enhances the pH sensitivity of the cytochrome *b*_6_*f* complex, partially restored photosystem I oxidation and alleviated photosystem I photodamage in the *pgr5* background. However, excessively strong photosynthetic control limits electron transport at relatively low light intensities. To investigate whether a milder enhancement of photosynthetic control can protect photosystem I without compromising photosynthetic performance, we introduced a series of amino acid substitutions into the Rieske subunit of the cytochrome *b*_6_*f* complex using Target-activation-induced cytidine deaminase (AID)-mediated base editing. Among these, the E143K mutation partially oxidized photosystem I and improved photosynthetic induction in the *pgr5-2* background more effectively than the *pgr1* mutation. Although the E143K mutation had little effect on electron transport parameters in the wild-type background, it significantly reduced the proton motive force. The unexpected reduction in proton motive force suggests that moderately enhanced photosynthetic control can be accommodated without major impairment of photosynthetic electron transport.

## Introduction

Absorption of excess light energy that cannot be used for CO_2_ fixation can damage the photosynthetic apparatus through the generation of reactive oxygen species. To prevent such photodamage, plants have developed multiple regulatory mechanisms that downregulate light energy utilization. One of the most important strategies is the thermal dissipation of excess excitation energy in the antennae of photosystem II (PSII), which is detected as nonphotochemical quenching (NPQ) of chlorophyll fluorescence (van Amerongen and Croce 2025). PSII was long considered the primary target of photodamage; however, damaged PSII can be repaired efficiently (Komenda et al. 2024). In contrast, photodamage to photosystem I (PSI) was thought to occur only under limited conditions (Sonoike 2025). Recent studies have shown that PSI is generally resistant to photodamage because it is protected by multiple mechanisms (Shikanai 2024). Such tight protection is essential because PSI photodamage is particularly harmful, as plants lack an efficient repair system for damaged PSI.

Cyclic electron transport (CET) around PSI was originally discovered by Arnon et al. (1954) as a mechanism for ATP generation in chloroplasts without concomitant water splitting at PSII or reduction of NADP^+^. This classical CET pathway is sensitive to antimycin A (Tagawa et al. 1963) and was later rediscovered through the identification of the Arabidopsis (*Arabidopsis thaliana*) *proton gradient regulation 5* (*pgr5*) mutant, which is defective in full NPQ induction (Munekage et al. 2002; Sugimoto et al. 2013). In the *pgr5* mutant, the PSI reaction center chlorophyll pair P700 remains highly reduced, leading to severe PSI photodamage.

In the light, PGR5-dependent CET oxidizes P700 through 2 mechanisms (Zhou et al. 2022). First, it promotes acidification of the thylakoid lumen, thereby downregulating the activity of the cytochrome *b*_6_*f* (Cyt *b*_6_*f*) complex (Suorsa et al. 2012). This process, termed photosynthetic control, is essential for protecting PSI, particularly under fluctuating light conditions (Degen and Johnson 2024). At the molecular level, photosynthetic control is thought to be regulated by pH-dependent modulation of the Cyt *b*_6_*f* complex. Despite its physiological importance and relatively early discovery (Rumberg and Siggel 1969; Tikhonov et al. 1981; Nishio and Whitmarsh 1993), it remains unclear how the Cyt *b*_6_*f* complex senses luminal acidification to downregulate its activity. A mechanistic model, based on studies of the mitochondrial cytochrome *bc*_1_ complex (Covián and Moreno-Sánchez 2001), proposes that the His178 residue of the Rieske subunit, which ligands the iron–sulfur (FeS) cluster, becomes protonated under low luminal pH, thereby contributing to downregulation of Cyt *b*_6_*f* activity (Finazzi et al. 2016; Malone et al. 2021). The numbering refers to the full-length Arabidopsis protein, including the transit peptide (Q9ZR03), and corresponds to His126 in the mature spinach protein. The p*K*_a_ of this residue depends on the redox state of the FeS cluster (Tikhonov 2014). Notably, the Arabidopsis *pgr1* mutation from Pro194 to Leu (P194L), described in detail below, is located in close proximity to His178 and may alter its p*K*_a_ (Munekage et al. 2001).

Second, in parallel with photosynthetic control on the donor side, PGR5-dependent CET also contributes to P700 oxidation by preventing excessive accumulation of electrons on the acceptor side of PSI. In many photosynthetic organisms, this acceptor-side regulation is mediated by flavodiiron proteins (Flv), which reduce O_2_ to H_2_O by accepting electrons from NADPH or ferredoxin (Vicente et al. 2002; Sétif et al. 2020). Although Flv proteins are conserved from cyanobacteria to gymnosperms, they were lost during the early evolution of angiosperms (Yamamoto et al. 2016; Ilik et al. 2017). In the absence of Flv, angiosperms rely on PGR5-dependent CET to redistribute excess electrons from the PSI acceptor side into the plastoquinone (PQ) pool, thereby maintaining P700 in an oxidized state (Zhou et al. 2022).

Because angiosperms lack Flv, donor-side regulation of PSI via photosynthetic control is especially important in these plants. However, direct assessment of the physiological significance of photosynthetic control has been difficult, as mutants with selectively attenuated photosynthetic control have not been identified. An alternative strategy to evaluate its function involves overexpression of the *KEA3* gene, which encodes a thylakoid-localized H^+^/K^+^ antiporter (Armbruster et al. 2014; Kunz et al. 2014). Photosynthetic control is induced by luminal acidification, but KEA3 shifts components of the proton motive force (pmf) from the proton concentration gradient (ΔpH) to the membrane potential (ΔΨ). Consequently, KEA3 overaccumulation reduces luminal acidification and attenuates photosynthetic control (Armbruster et al. 2016). This attenuation leads to an increased reduction of P700 under illumination. The effect is even more pronounced in the *disturbed proton gradient regulation* (*dpgr*) mutant form of KEA3, which likely causes excessive proton leakage from the thylakoid lumen (Wang et al. 2017). Notably, the transgenic lines overexpressing the *dpgr-*type KEA3 phenocopied the *pgr5* mutant, which was unable to survive under fluctuating light conditions.

In contrast, the Arabidopsis *pgr1* mutant exhibits enhanced photosynthetic control (Jahns et al. 2002). This mutant was originally isolated based on its impaired NPQ induction (Munekage et al. 2001) and carries a P194L substitution in the Rieske subunit of the Cyt *b*_6_*f* complex. Although this mutation does not affect the stability of the Rieske protein, it alters the pH sensitivity of the Cyt *b*_6_*f* complex. Owing to its strong photosynthetic control, introduction of the *pgr1* mutation partially restored P700 oxidation in the *pgr5* background, in which luminal acidification is compromised due to impaired CET (Yamamoto and Shikanai 2019). As a result, the *pgr1 pgr5* double mutant regained PSI resistance to fluctuating light at levels comparable to the wild type (WT). Moreover, in the presence of functional PGR5, PSI in the *pgr1* mutant is even more resistant to fluctuating light.

Despite conferring enhanced PSI protection against fluctuating light, the *pgr1* mutation renders PSII more susceptible to constant high light, and electron transport becomes saturated at lower light intensities (Munekage et al. 2001). These observations raise several important questions: How has the extent of photosynthetic control been optimized during the evolution of land plants? Can its magnitude be artificially modified to re-optimize photosynthetic performance under specific growth environments? To address these questions, we screened Arabidopsis mutants with moderately enhanced photosynthetic control activity but without deleterious secondary effects.

## Results

### Generation of 23 new *pgr1* alleles

To isolate Arabidopsis mutants with moderately altered photosynthetic control, we focused on the Rieske subunit of the Cyt *b*_6_*f* complex. We introduced a series of site-directed mutations into the luminal region of the Rieske protein using the Target-activation-induced cytidine deaminase (AID) base editing system (Nishida et al. 2016). Instead of introducing insertions and deletions via CRISPR-Cas9-mediated DNA double-strand breaks, which typically disrupt genes, base editing mediated by deaminases can introduce specific base substitutions that enable detailed functional genetics. The Target-AID system employs PmCDA1 fused to nickase Cas9 and efficiently induces cytosine substitutions within a 3- to 5-base window at the distal end of target sequences relative to the PAM sequence, as demonstrated in plants such as rice (*Oryza sativa*) and tomato (*Solanum lycopersicum*; Shimatani et al. 2017).

In Arabidopsis, the *petC* gene encodes a protein of 229 amino acids (aa), including its plastid-targeting sequence, and the C-terminal 127 aa (positions Thr103 to Ser229) comprise the luminal region. Based on the requirement of the PAM sequence (NGG-3′) and the editing window of Target-AID, 44 residues are estimated to be editable. We further prioritized residues that are highly conserved across land plants but not conserved in algae or cyanobacteria (Figure S8), assuming that such sites may underlie lineage-specific optimization of photosynthetic control in terrestrial environments. In contrast, mutations in residues conserved among all phototrophs were avoided because alterations at such positions could have excessively strong effects, as seen for the original *pgr1* mutant (Munekage et al. 2001).

The mutagenesis was introduced into both the Arabidopsis WT and the *pgr5-2* mutant backgrounds. The *pgr5-2* mutant is a weak *pgr5* allele in which the PGR5 protein accumulates to ∼50% of the WT level (Yamamoto and Shikanai 2019). Consistent with the reduced activity of PGR5-dependent CET, Y(I) in *pgr5-2* is intermediate between that of WT and *pgr5-1*, resulting in a mild elevation of the luminal pH. Accordingly, under constant low light, growth is not impaired even in double mutants carrying the *pgr5-2* allele together with a defect in the NDH pathway (Nakano et al. 2019). We reasoned that screening in the *pgr5-2* background might enable the identification of mutants showing even subtler increases in photosynthetic control.

Twenty target sites were selected (Table S1), and the corresponding editing constructs were transformed, yielding 237 independent T_1_ plants in total. Subsequently, 954 transgene-free T_2_ plants were grown on soil. The absence of the transgene was confirmed by loss of seed fluorescence, as the transgene product was fused to a TagRFP marker targeting oil bodies in seeds (Tsutsui and Higashiyama 2017). Genomic DNA from each T_2_ plant was extracted and subjected to PCR amplification of the targeted *petC* regions. This analysis identified 45 independent lines carrying 23 distinct novel mutations in *petC,* with 12 in WT, 8 in *pgr5-2*, and 3 in both backgrounds (Figure S1 and Table S1). Most mutant lines grew comparably to WT under soil-grown conditions. However, the R187K mutant in the WT background exhibited noticeably reduced growth. In addition, the double mutant carrying L119V and H122D substitutions, as well as the T128I single mutant in the WT background, showed an albino phenotype on MS medium supplemented with 2% sucrose.

### The E143K mutant showed partial oxidation of PSI in the *pgr5-2* background

Oxidation of the PSI special pair chlorophylls (P700) was monitored as absorbance changes at 810 nm using a Dual-PAM system. The Y(ND) parameter represents the fraction of PSI that remains oxidized P700 under actinic light (AL), which primarily reflects limitations on electron transfer through the Cyt *b*_6_*f* complex (photosynthetic control). As a secondary screening step, we measured Y(ND) in all mutants under AL at 100 µmol photons m^−2^ s^−1^. No mutants exhibited a reduced Y(ND) value, indicating no weakened photosynthetic control. In contrast, the E143K mutant displayed an increased Y(ND) level in the *pgr5-2* background, suggesting enhanced photosynthetic control.

The E143K allele was identified in the *pgr5-2* background (hereafter E143K *pgr5-2*) and the WT background (hereafter E143K). Both E143K *pgr5-2* and E143K plants grew similarly to WT under standard growth-chamber conditions (Figure S2). To assess whether the E143K mutation affects the in vivo stability of the Cyt *b*_6_*f* complex, we performed protein blot analyses using antibodies against major thylakoid membrane proteins. Across all genotypes, including *pgr1*, subunit abundance was comparable, indicating that the E143K mutation does not compromise Cyt *b*_6_*f* stability, as observed with the *pgr1* mutation (Figure S3).

**Figure 1:**
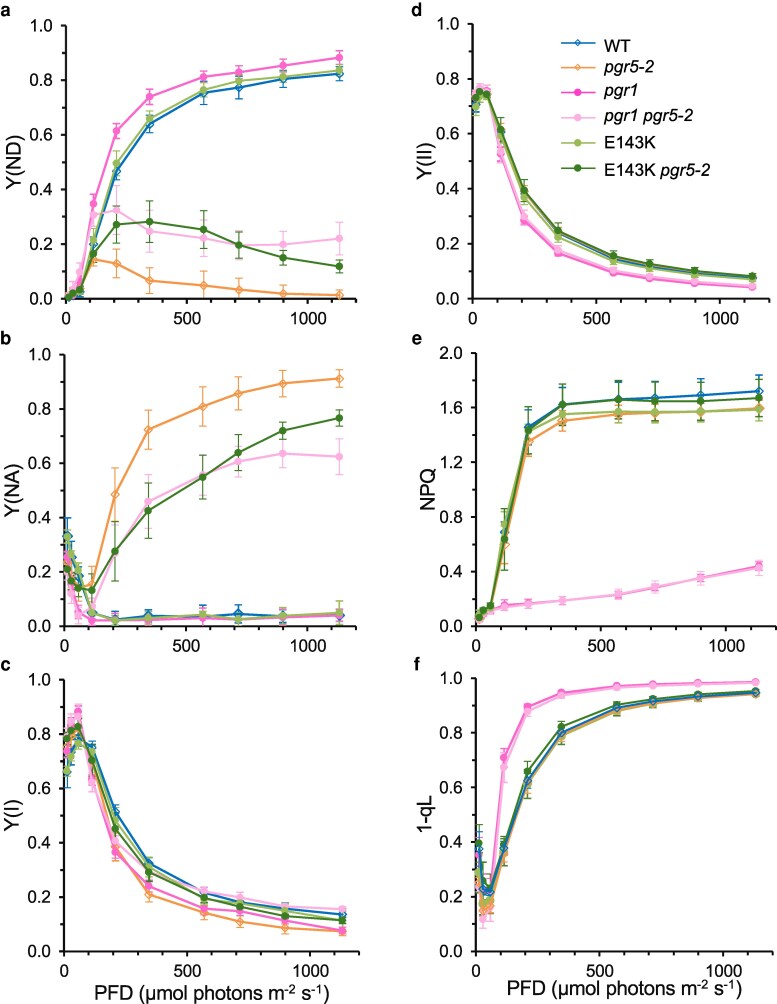
Light-intensity dependence of P700 and chlorophyll fluorescence parameters during steady-state photosynthesis. The parameters a) Y(ND), b) Y(NA), c) Y(I), d) Y(II), e) NPQ, and f) 1 − qL were measured in detached leaves of WT, *pgr5-2*, *pgr1*, E143K, *pgr1 pgr5-2*, and E143K *pgr5-2* plants. Data are presented as means ± Sd (*n* = 6, biological replicates). Statistical analyses were conducted using the Tukey–Kramer test and are summarized in Table S2.

Figure 1a shows the light-intensity dependence of Y(ND). In the *pgr5-2* mutant, P700 remained highly reduced because of the elevated luminal pH. Consistent with a previous report (Yamamoto and Shikanai 2019), introducing the *pgr1* mutation into the *pgr5-2* background partially restored P700 oxidation. A similar effect was observed in the E143K *pgr5-2* mutant. In the WT background, the *pgr1* mutation increased P700 oxidation compared with WT plants (Fig. 1a), whereas the E143K mutation had no detectable effect on P700 redox state.

The Y(NA) and Y(I) parameters reflect the fractions of reduced PSI that are unable or able to transfer electrons to downstream acceptors, respectively (Fig. 1b and c). In the *pgr5-2* mutant, Y(NA) was markedly elevated due to impaired CET (Fig. 1b). This acceptor-side limitation arises not only from weakened photosynthetic control but also from diminished acceptor-side regulation, which helps oxidize P700 by pulling electrons from PSI (Yamamoto and Shikanai 2019; Zhou et al. 2022). In contrast, Y(NA) levels were substantially reduced in the *pgr1* and *pgr1 pgr5-2* mutants, even below WT levels at low light intensities below 58 µmol photons m^−2^ s^−1^, reflecting strong photosynthetic control that operates even at neutral luminal pH. Introduction of both the *pgr1* and E143K mutations into the *pgr5-2* background partially alleviated the elevated Y(NA) phenotype at higher light intensities.

Y(I) was lower in both the *pgr1* and *pgr5-2* mutants at light intensities above 209 µmol photons m^−2^ s^−1^, but for distinct physiological reasons (Fig. 1c). In the *pgr1* mutant, strong photosynthetic control restricted PSI photochemistry, consistent with its high Y(ND). In the *pgr5-2* mutant, however, decreased Y(I) likely results from acceptor-side limitation that restricts electron flow downstream of PSI.

Chlorophyll fluorescence parameters reflecting PSII activity were consistent with the changes observed in PSI. Y(II) was reduced at high-light intensities in the *pgr1* background, which is expected under strong photosynthetic control (Fig. 1d). NPQ levels were extremely low in both the *pgr1* and *pgr1 pgr5-2* mutants (Fig. 1e). In addition, the PQ pool was more reduced in the *pgr1* background (Fig. 1f), indicating enhanced electron accumulation upstream of the Cyt *b*_6_*f* complex.

### Photosynthetic control is specifically affected in the E143K mutant

**Figure 2:**
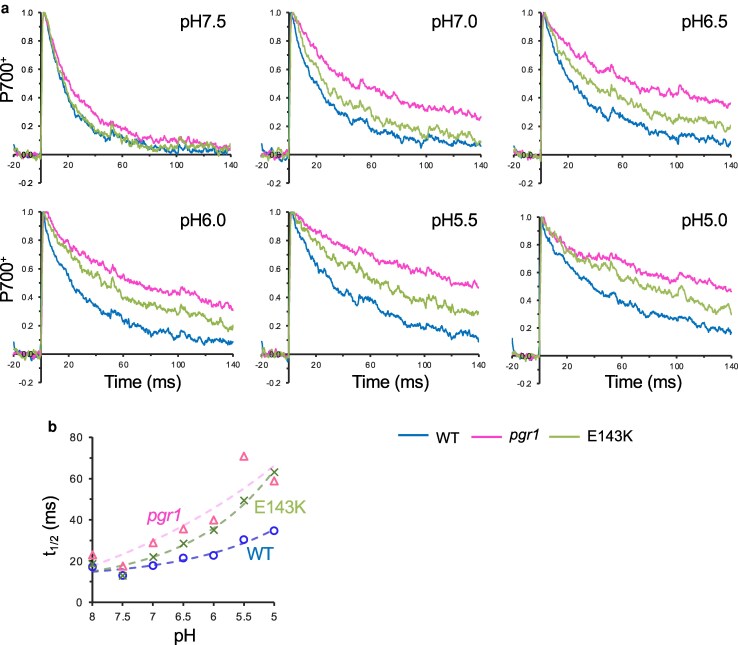
pH dependence of linear electron transport. a) A single turnover flash (5 µs, 20,000 µmol photons m^−2^ s^−1^) was applied to transiently oxidize P700, and the subsequent re-reduction driven through the Cyt *b*_6_*f* complex was monitored. Signals were recorded with a time resolution of 10 µs per point, and 20 points were averaged to obtain a mean value over 200 µs. Forty signals were averaged under repetitive excitation at 2 s intervals. Upward deflections indicate P700 oxidation. Signal amplitudes are presented relative to the preflash absorption level (set to 0) and the maximal P700^+^ level (set to 100). b) The decay curves were fitted with a single-exponential function, and the resulting half-time (*t*_1/2_) values were plotted against pH (WT, circles; E143K, crosses; and *pgr1*, triangles). Dashed lines indicate the fitted trends for each genotype.

General reductions in Q-cycle activity can also contribute to P700 oxidation under illumination (Yamazaki et al. 2004). However, the abundance of the Cyt *b*_6_*f* complex was unchanged in the E143K mutant (Figure S3), indicating that the enhanced P700 oxidation cannot be explained by a decreased level of the complex. Previously, we demonstrated enhanced pH sensitivity of photosynthetic control in the *pgr1* mutant by examining the pH dependence of linear electron transport to PSI in isolated thylakoids (Jahns et al. 2002). To compare this behavior with the E143K mutant, we isolated thylakoid membranes from WT, *pgr1*, and E143K plants. A flash was applied to transiently oxidize P700, and the rate of re-reduction by electron flow through the Cyt *b*_6_*f* complex was monitored in buffers of varying pH (Fig. 2a). At pH 7.5, all 3 genotypes exhibited similar P700 reduction rates based on half-times (Fig. 2b), indicating that the E143K mutation does not alter the basic Q-cycle activity of the Cyt *b*_6_*f* complex, consistent with its unchanged protein abundance (Figure S3). In contrast, at lower pH values, both the *pgr1* and E143K mutants showed slower P700 reduction than WT, indicating increased pH sensitivity of the Q cycle. The effect was milder in the E143K mutant compared with the *pgr1* mutant.

### The E143K mutation is superior to the *pgr1* mutation for oxidizing P700 during the induction of photosynthesis at moderate light intensity

**Figure 3:**
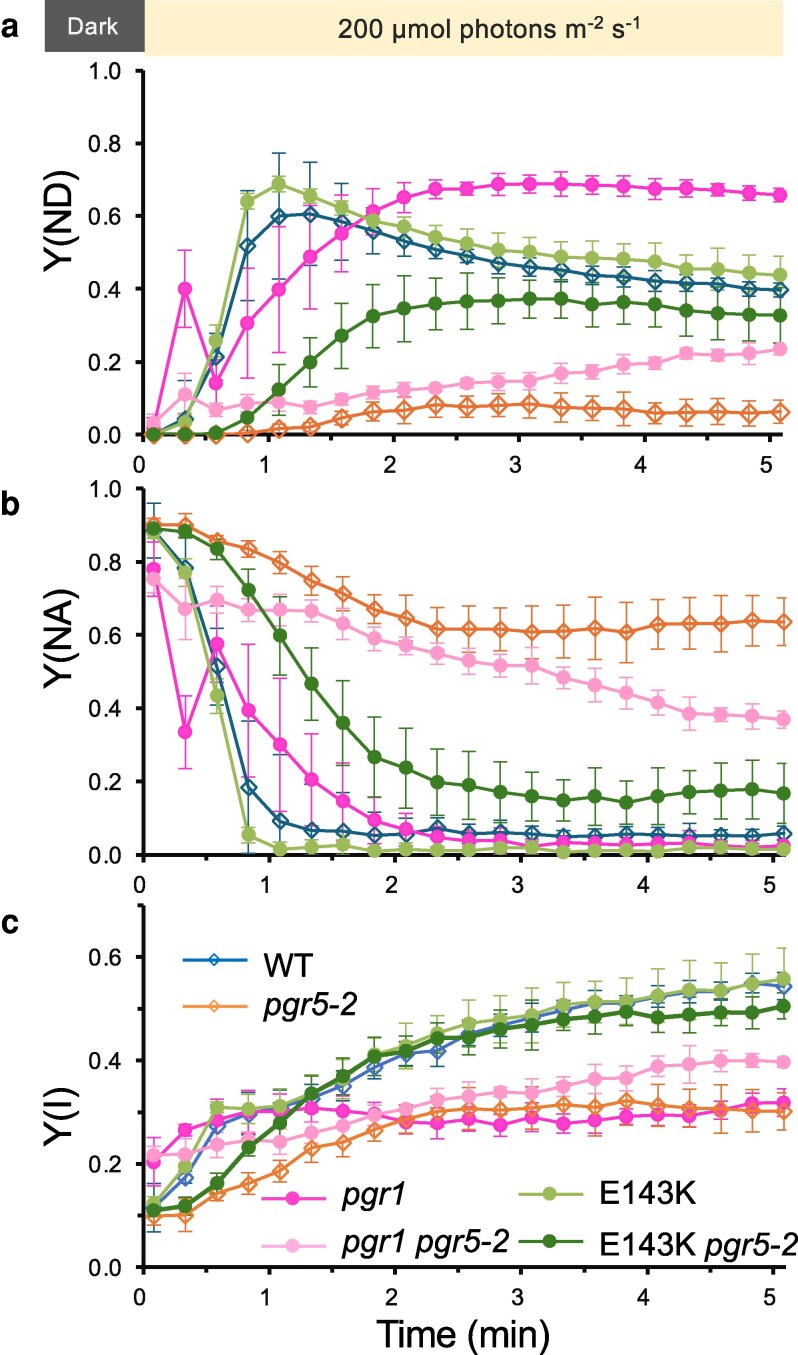
Induction of electron transport. Photosynthesis was induced by nonsaturating actinic light (200 µmol photons m^−2^ s^−1^). The parameters a) Y(ND), b) Y(NA), and c) Y(I) were measured using a Dual-PAM-100 system. Detached leaves from dark-adapted plants were exposed to actinic light. Statistical analyses were conducted using the Tukey–Kramer test and are summarized in Table S3.

To evaluate the impact of enhanced photosynthetic control in vivo, we monitored the time courses of photosynthetic parameters during induction at a nonsaturating light intensity (200 µmol photons m^−2^ s^−1^; Fig. 3). In the *pgr1* mutant, pronounced oscillations in P700 oxidation were observed within the first minute (Fig. 3a). These oscillations likely reflect transient increases in luminal pH caused by excessively strong photosynthetic control. As a consequence, the induction of Y(II) was delayed in the *pgr1* mutant (Figure S4b). In contrast, the E143K mutant behaved similarly to WT during the induction process.

In the *pgr5-2* background, however, the E143K mutation partially restored PSI oxidation >1 min after the induction of photosynthesis, indicating a mild enhancement of photosynthetic control (Fig. 3a). The induction curve of E143K *pgr5-2* retained the overall shape of the *pgr5-2* curve but was vertically expanded, suggesting that the E143K mutation strengthens the weak photosynthetic control that still operates under the higher luminal pH characteristic of the *pgr5-2* background. The *pgr1 pgr5-2* mutant showed a distinct behavior: A small degree of P700 oxidation occurred at low light intensities, probably due to the high pH sensitivity of the Cyt *b*_6_*f* complex, but the time required to reach steady-state oxidation was considerably longer. This likely accounts for the apparent discrepancy in P700 oxidation between steady-state and induction measurements at the same light intensity (Figs. 1a and 3a).

The Y(NA) parameter mirrored the changes observed in Y(ND) (Fig. 3b). Despite its weaker pH sensitivity compared with the *pgr1* mutation, the E143K mutant was more effective than the *pgr1* mutation at suppressing the buildup of potentially harmful Y(NA) during induction in the *pgr5-2* background. In the E143K *pgr5-2* mutant, Y(I) levels were initially low, similar to the *pgr5-2* mutant, due to high Y(NA) during the first minute of induction (Fig. 3b and c). Thereafter, Y(I) gradually returned to WT levels, accompanied by a reduction in Y(NA) driven by the E143K-dependent enhancement of photosynthetic control. This recovery pattern did not occur in the *pgr1 pgr5-2* mutant, likely because the overly strong photosynthetic control associated with the *pgr1* mutation further elevated the already high luminal pH in the *pgr5-2* background. This interpretation is consistent with the extremely low NPQ phenotype observed in the *pgr1* mutant background (*pgr1* and *pgr1 pgr5-2* in Figure S4a).

Target-AID enabled the identification of 2 additional independent alleles carrying distinct aa substitutions at the same E143 position, E143D and E143Q (Figure S1). To evaluate the effects of these substitutions, induction of photosynthesis was analyzed at the same light intensity (200 µmol photons m^−2^ s^−1^) in plants carrying each allele in the *pgr5-2* mutant background (Figure S5). Similar to the E143K allele, both E143D and E143Q partially restored P700 oxidation in the *pgr5-2* background, although their effects were weaker than those observed for E143K. The physicochemical properties of the residue at position 143, rather than charge alone, contribute to the modulation of photosynthetic control.

### The E143K mutation likely enhances photosynthetic control independently of the *pgr1*-dependent mechanism

Figure S6a shows the position of E143 in the luminal region of the Rieske subunit. Unlike the *pgr1* mutation, which is located near H178, a residue proposed to sense luminal pH (Jahns et al. 2002; Malone et al. 2021), E143 resides in a more distant region of the luminal domain. Structural analysis of the spinach Rieske protein using PyMOL 3.1 (https://www.pymol.org) predicted a potential hydrogen bond between E143 and T147 in a single Rieske subunit of the complex in 6QRF (Figure S6b). However, this interaction was not observed in other structures (7ZYV and 7QRM). Thus, the structural basis by which the E143K mutation affects pH-dependent regulation of the Q-cycle remains unclear. How, then, does the E143K mutation influence pH-dependent regulation of the Q-cycle? To address this question, we introduced the E143K mutation into the *pgr1* background using the Target-AID strategy, generating plants carrying both mutations within the same gene.

**Figure 4:**
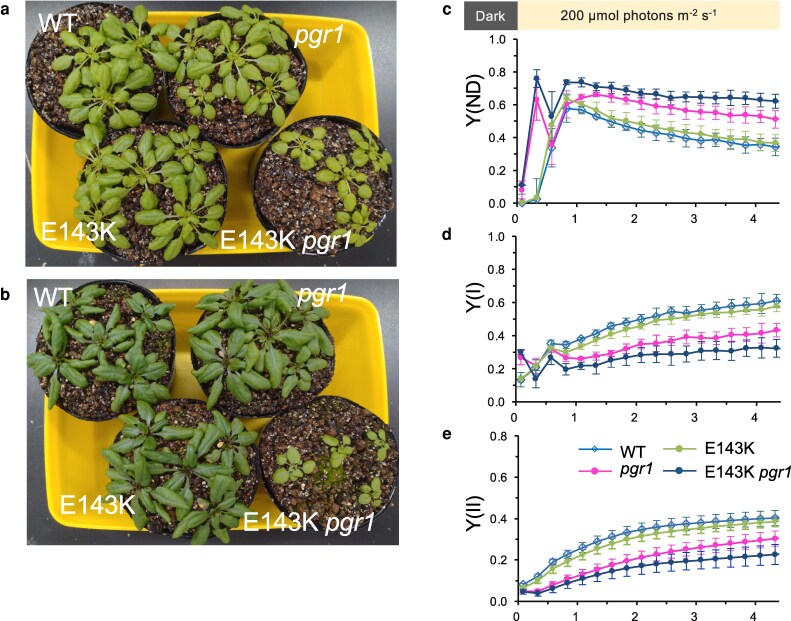
Delayed growth of the E143K *pgr1* mutant carrying both mutations in the *petC* gene (a and b). The growth phenotype was more pronounced under higher light intensity (120 µmol photons m^−2^ s^−1^) and long-day conditions (16 h photoperiod) (b) than under standard growth conditions (70 µmol photons m^−2^ s^−1^, 8 h photoperiod) (a). c to e) Induction of electron transport was analyzed using plants grown under the standard conditions shown in (a). Photosynthesis was induced by nonsaturating actinic light (200 µmol photons m^−2^ s^−1^). The parameters c) Y(ND), d) Y(NA), and e) Y(I) were measured using a Dual-PAM-100 system. Detached leaves from dark-adapted plants were exposed to actinic light. Statistical analyses were conducted using the Tukey–Kramer test and are summarized in Table S5.

Although every single mutant grew at rates comparable to WT plants, the double mutant showed delayed growth in soil, especially at a higher light intensity of 120 µmol photons m^−2^ s^−1^ (Fig. 4a and b). Figure 4c to e shows the induction kinetics of photosynthetic parameters under the same light conditions as in Fig. 3. The double mutant exhibited oscillations in Y(ND), similar to those observed in the *pgr1* mutant. However, its steady-state level of P700 oxidation was higher than in either single mutant. Thus, the E143K mutation synergistically enhances the *pgr1* phenotype. Consistent with the behaviors of the individual mutants (Fig. 3), these results suggest that the E143K and *pgr1* mutations enhance photosynthetic control through distinct molecular mechanisms (see Discussion).

### The E143K mutation had a limited effect on PSI oxidation under fluctuating light

**Figure 5:**
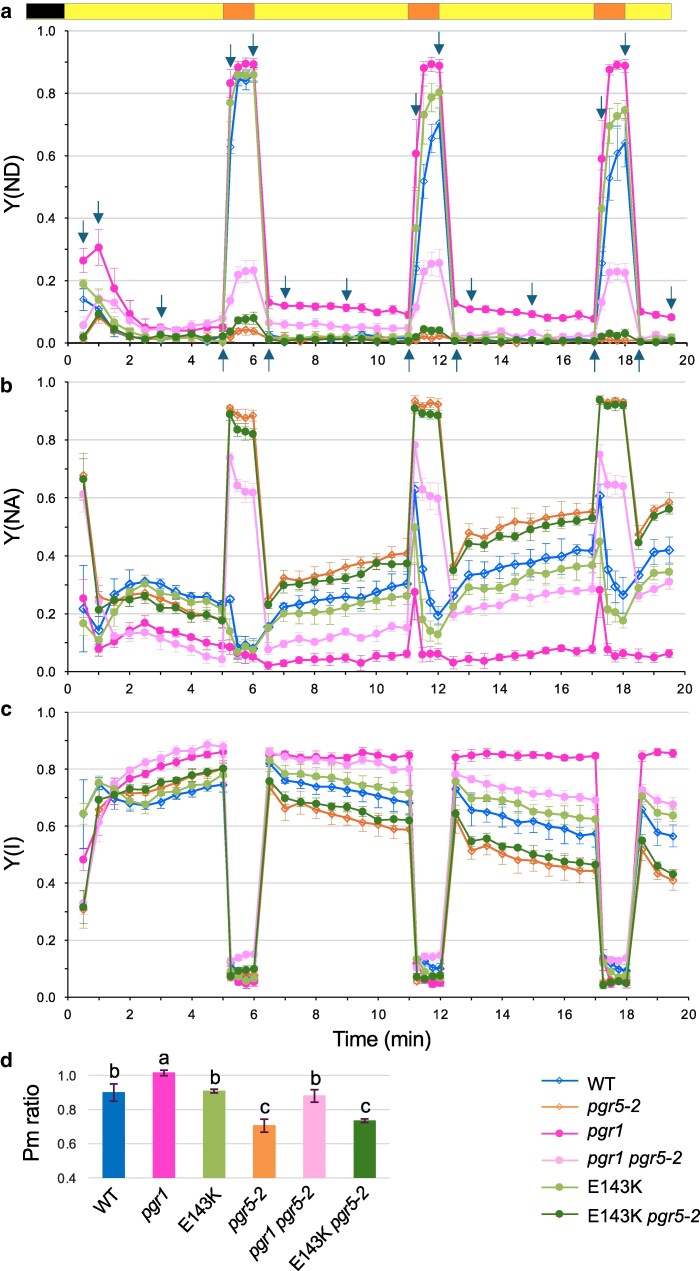
P700 parameters during the fluctuating light condition consisting of 5 min LL (longer bars) of 50 μmol photons m^−2^ s^−1^ and 1 min HL (shorter bars) of 1,510 μmol photons m^−2^ s^−1^. A black bar represents the adaptation to the dark. Y(ND) (a), Y(NA) (b), and Y(I) (c) were analyzed in detached leaves from each genotype indicated. Data represent mean ± Sd (*n* = 5, biological replicates). Statistical analyses were conducted using the Tukey–Kramer test and are summarized in Table S6. Vertical arrows indicate time points used for the statistical analyses. d) Evaluation of PSI photodamage during the fluctuating light condition. The Pm levels were determined before and after exposure to the fluctuating light. The ratio (after/before) is shown as a mean of 5 biological replicates. Error bars represent Sd. Different letters indicate statistically significant differences by the Tukey–Kramer test (*P* < 0.05).

Enhanced photosynthetic control is known to confer PSI resistance to fluctuating light intensity (Yamamoto and Shikanai 2019). To assess the contribution of the E143K mutation to PSI resistance under fluctuating light, the mutant lines were exposed to the fluctuating light consisting of low light (50 µmol photons m^−2^ s^−1^, 5 min) and high light (1,510 µmol photons m^−2^ s^−1^, 1 min), and this cycle was repeated 3 times (Fig. 5). As observed under constant light conditions (Fig. 1a), the Y(ND) level was higher during the first high-light period in the E143K *pgr5-2* mutant than in the *pgr5-2* mutant (Fig. 5a and Table S6). However, the difference was not significant during the second and third high-light periods. This is contrasting with the *pgr1* mutation, which increased the Y(ND) level throughout the entire course of the experiment. In contrast, in the WT background, the Y(ND) level was higher in the E143K mutant than in WT during the second and third high-light periods, but not during the first high-light period (Fig. 5a). No clear phenotype of the E143K mutation was detected in the chlorophyll fluorescence parameters (Figure S7 and Table S6).

After 3 cycles of fluctuating light, PSI photodamage was evaluated as the ratio of Pm before and after the fluctuating light treatment. Unlike the *pgr1* mutation, which conferred PSI resistance to fluctuating light in both the WT and *pgr5-2* backgrounds, the E143K mutation did not improve PSI resistance in either background (Fig. 5d). Although the E143K mutation mildly enhanced photosynthetic control, its effect was not detectable under these short-term fluctuating light conditions.

### Enhanced photosynthetic control is accompanied by lower pmf

The E143K mutant phenotype was initially identified in the *pgr5-2* background, where it partially compensated for the lack of CET by promoting P700 oxidation. In contrast, the E143K mutant displayed no obvious phenotype in the WT background in vivo (Figs. 1 and 3). To further characterize its physiological impact, we examined how the pmf responds to changes in light intensity. The pmf is the electrochemical gradient of protons across the thylakoid membrane generated by photosynthetic electron transport and drives ATP synthase rotation.

**Figure 6:**
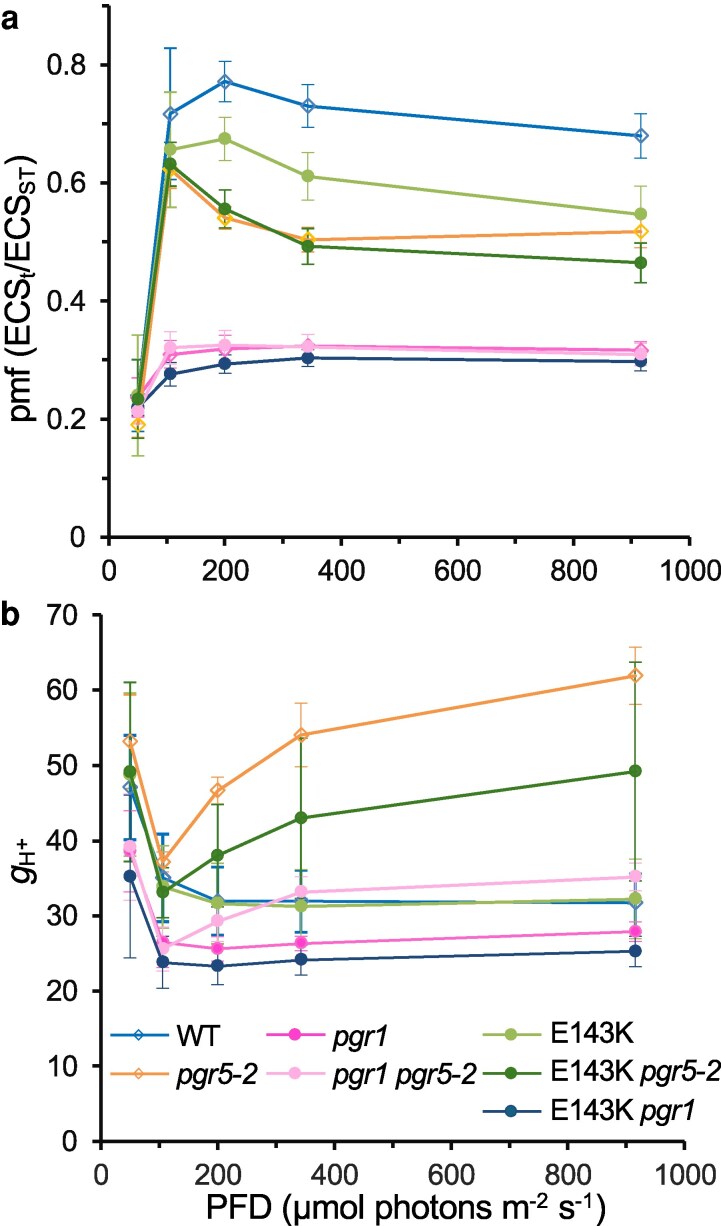
Light-intensity dependence of ECS parameters. a) The magnitude of the pmf generated in the light was calculated as ECS_t_/ECS_ST_. b) The proton conductivity parameter (*g*_H_^+^) was determined in the same analysis. Data are presented as means ± Sd (*n* = 5, biological replicates). Statistical analyses were conducted using the Tukey–Kramer test and are summarized in Table S7.

Unexpectedly, the E143K mutant in the WT background showed a markedly reduced pmf at light intensities above 200 µmol photons m^−2^ s^−1^, with values intermediate between WT and *pgr5-2* (Fig. 6a). Because the E143K mutation does not alter the rate of electron transport, the reduced pmf is unlikely to be explained by a limitation in electron flow, although the accuracy of Y(I) as a proxy for PSI activity should be interpreted cautiously.

We next tested whether increased proton conductivity through ATP synthase could account for the reduced pmf by monitoring the *g*_H_^+^ parameter (Fig. 6b). Consistent with previous reports (Avenson et al. 2005; Wang et al. 2015), *g*_H_^+^ was elevated in the *pgr5-2* mutant at high-light intensities. We previously explained this phenotype by 2 possibilities: (i) The high luminal pH in the *pgr5-2* mutant does not slow the Q-cycle, resulting in faster consumption of reducing equivalents and electrochromic shift (ECS) relaxation during dark pulses, and (ii) the absence of PGR5-dependent PQ reduction may accelerate oxidation of the Q-cycle carrier (Yamamoto and Shikanai 2020). The reduced *g*_H_^+^ in the *pgr1* mutant, as well as the intermediate levels in the double mutants, can be interpreted using the same rationale. Notably, the *g*_H_^+^ level of the E143K mutant was similar to that of WT. Although the slower Q-cycle activity associated with E143K may influence *g*_H_^+^, we found no evidence that changes in proton conductivity contribute to the reduced pmf phenotype in this mutant.

Thus, the mechanism underlying the low pmf in the E143K mutant remains unresolved. Nevertheless, the association between enhanced photosynthetic control and reduced pmf suggests that a lower pmf may be required to balance electron transport and maintain optimal photosynthetic performance when photosynthetic control is upregulated. We will discuss this possibility further below.

## Discussion

The E143K mutant provided a useful tool for examining how a mild enhancement of photosynthetic control affects overall photosynthetic performance. Comparison with the previously identified *pgr1* mutant further elucidated how the Cyt *b*_6_*f* complex fine-tunes photoprotective regulation. It remains unclear how the Cyt *b*_6_*f* complex senses luminal acidification and subsequently downregulates its activity. As outlined in the Introduction, one proposed model suggests that protonation of His178, an aa ligating the 2Fe-2S ISP cluster, serves as a pH-sensing event (Finazzi et al. 2016; Malone et al. 2021). Because the *pgr1* mutation is located adjacent to His178, it may alter its p*K*_a_, thereby shifting the pH threshold for photosynthetic control (Jahns et al. 2002). In contrast, E143 resides in a distant region of the luminal domain and is unlikely to modify the p*K*_a_ of His178 (Figure S6a). Instead, the E143K mutation may influence conformational changes in the Rieske protein that occur upon protonation, thereby amplifying the output of photosynthetic control rather than altering pH sensitivity itself. Together, these findings support a model in which the E143K mutation enhances photosynthetic control through a mechanism separate from that of the *pgr1* mutant.

As we selected sites preferentially conserved in land plants, E143 is not highly conserved in algae (Figure S8). Interestingly, lysine is found at this position in some green algae and diatoms. It is unclear whether the lysine residue in these organisms enhances the extent of photosynthetic control as in Arabidopsis, since it may depend on the full structure of the Rieske protein in each species. In land plants, however, glutamate is conserved at this site.

The E143K mutant phenotype was initially identified in the *pgr5-2* background, where it partially compensated for the loss of CET by promoting P700 oxidation. In contrast, the E143K mutation produced no obvious phenotype in the WT background in vivo (Figs. 1 and 3), except for the enhanced oxidation of PSI under fluctuating light conditions (Fig. 5a). However, our analysis revealed that the pmf level was reduced in the E143K mutant, and at 900 µmol photons m^−2^ s^−1^ it approached that of the *pgr5-2* mutant (Fig. 6a). Despite this low pmf, the 2 mutants displayed markedly different P700 redox phenotypes: whereas P700 remained highly reduced in the *pgr5-2* mutant, it was oxidized to WT levels in the E143K mutant (Fig. 1a and b). These contrasting phenotypes indicate that 2 mechanisms contribute to P700 oxidation in the E143K mutant. First, E143K-dependent enhancement of photosynthetic control allows Cyt *b*_6_*f* downregulation to operate even under conditions of elevated luminal pH, as observed in the E143K *pgr5-2* mutant (Fig. 1a). Second, because PGR5-dependent CET remains functional in the E143K mutant, the acceptor-side regulation can fully oxidize P700, as proposed previously (Zhou et al. 2022). Together, these mechanisms enable the E143K mutant to oxidize P700 effectively even when pmf is reduced, distinguishing it from the *pgr5-2* mutant.

Although the mechanism underlying the low-pmf phenotype in the E143K mutant remains unresolved, the present results allow several tentative interpretations. One possibility is that the E143K mutation affects a proton egress pathway from the *Q*_o_ site to the bulk lumen. The charge reversal from E to K on the surface of the Rieske large subdomain could create a local electrostatic barrier to proton release. In principle, this could explain both the reduction in bulk pmf and the enhancement of photosynthetic control, if protons were retained near the regulatory site of the Cyt *b*_6_*f* complex even when the overall pmf remained modest. However, because the E143D and E143Q substitutions also affected the degree of photosynthetic control, at least in the *pgr5-2* background (Figure S5), the phenotype may not be explained simply by the surface charge at this position.

A second possibility is that the enhanced photosynthetic control itself slightly restricts electron transport, thereby lowering pmf. This interpretation is consistent with our previous view of the *pgr1* phenotype, in which strong photosynthetic control suppresses both Y(I) and Y(II), restricts electron transport, and is accompanied by a marked reduction in pmf (Munekage et al. 2001). In the present study, however, this explanation is not directly supported by the measurements, because neither the electron transport parameters estimated from Y(II) and Y(I) nor the proton conductivity (*g*_H_^+^) showed clear alterations (Figs. 1c, d and 6b). Still, we cannot exclude the possibility that the E143K mutation causes a mild restriction of electron transport that is too subtle to be resolved in Y(I) or Y(II) but is nevertheless reflected in the pmf level.

A third possibility is not mutually exclusive with the second idea. The low pmf represents a compensatory response that helps maintain photosynthetic balance in the E143K background, rather than being a direct consequence of enhanced photosynthetic control. Even under conditions in which pmf was markedly reduced, parameters of photosynthetic electron transport remained largely unchanged in the E143K mutant (Figs. 1 and 3). This observation raises the possibility that a lower pmf may function as an adaptive adjustment. For example, if the E143K mutation promotes P700 oxidation more strongly than in the WT, maintenance of WT-level pmf might trigger additional feedback regulation, such as downregulation of CET (Okegawa et al. 2008). In this scenario, the low-pmf phenotype would reflect a secondary adjustment associated with the altered regulatory state rather than a direct mechanistic effect of the mutation itself. At present, the available data do not allow us to distinguish among these possibilities.

Based on the differences in phenotypes in the WT background (*pgr1* and E143K), the *pgr1* mutation appears to have a greater impact on photosynthetic control than the E143K mutation (Figs. 1 to 3). In the *pgr5-2* mutant background, however, the E143K mutation restored P700 oxidation more effectively than the *pgr1* mutation during the induction of photosynthesis at 200 µmol photons m^−2^ s^−1^ (Fig. 3a). To explain this discrepancy, it is necessary to consider the different mechanisms to enhance photosynthetic control between the 2 mutants, as discussed above. Although the underlying mechanism remains unresolved, photosynthesis in the E143K mutant appears to be rebalanced effectively at a lower pmf. In contrast, such rebalancing may not be achievable when photosynthetic control becomes excessively strong, as in the *pgr1* mutant. In the *pgr1* mutant, even under extremely low pmf, the strong photosynthetic control continues to restrict electron transport, delaying the attainment of steady-state P700 oxidation (Fig. 3a). This interpretation is supported by the observation that introducing the *pgr5-2* mutation into the *pgr1* background partially restored the induction of Y(II) (Figure S4b). Reducing photosynthetic control through the *pgr5-2* mutation alleviates the over-restriction imposed by the *pgr1* mutation, thereby allowing electron transport to recover. This supports the idea that the excessive strength of photosynthetic control in the *pgr1* mutant prevents rebalancing at low pmf, in clear contrast to the behavior of the E143K mutant.

Although the E143K mutant appears normal under growth-chamber conditions, its low pmf may not be advantageous in natural, fluctuating environments. Given that the E143 position is highly conserved among land plants, substituting this residue with lysine is unlikely to represent an evolutionarily viable means of enhancing photosynthetic control. Instead, the extent of photosynthetic regulation at this site may already have been optimized in land plants to balance protection and productivity under variable terrestrial light conditions.

## Materials and methods

### Plant materials and growth conditions

*Arabidopsis thaliana* (accession Columbia *gl1*) WT and mutant plants were grown in soil in a controlled growth chamber under a light intensity of 50 to 80 µmol photons m^−2^ s^−1^, a 16 h photoperiod, at 23 °C and 55% relative humidity. For electron transport analyses, plants were grown under short-day conditions (8 h photoperiod). Fully expanded leaves were used for all experiments.

### Introduction of site-directed mutations

The OLE1–TagRFP and single-guide RNA (sgRNA) expression cassette was excised from the pKIR1.1 vector (Tsutsui and Higashiyama 2017) by AscI–PmeI digestion and ligated into the pDicAID_nCas9-PmCDA_NptII_Della vector (Shimatani et al. 2017). Oligonucleotides containing the guide RNA sequences (Table S1) were annealed and inserted into the AarI site of the vector. The resulting plasmid was introduced into *Escherichia coli* for sequence verification and subsequently transferred into *Agrobacterium tumefaciens* strain C58C1 (pMP90). Arabidopsis WT, *pgr5-2*, and *pgr1* plants were independently transformed with each plasmid, and T_1_ seeds exhibiting TagRFP fluorescence were selected using a fluorescence microscope. The mutations were confirmed by sequencing the PCR products amplified with primers 5′-atggcgtcctcatccctttc-3′ (*petC*-fw1) and 5′-ggcaagaattcacagagtaattgcg-3′ (*petC*-rv). Primers 5′-gtaccttctttgttcctcctgg-3′ (*petC*-fw2) and *petC*-rv were used for sequencing. To eliminate the transgene, nonfluorescent T_2_ seeds were screened.

### Analysis of thylakoid membrane proteins

Chloroplasts were isolated from leaves of 4- to 5-wk-old plants, as described previously (Munekage et al. 2002). The isolated chloroplasts were osmotically ruptured in buffer containing 20 mm HEPES–KOH (pH 7.6), 5 mm MgCl_2_, and 2.5 mm EDTA. The insoluble fraction containing thylakoid membranes and envelope membranes was separated from the soluble fraction by centrifugation at 10,000 × *g* for 10 min at 4 °C. Chlorophyll concentration was determined as described previously (Porra et al. 1989). Proteins were solubilized in SDS–PAGE sample buffer. Thylakoid membrane proteins were separated by 12.5% (w/v) SDS–PAGE and electrotransferred onto PVDF membranes. Membranes were incubated with primary antibodies, and protein–antibody complexes were detected using the ECL Prime western blotting detection system (GE Healthcare). Chemiluminescence signals were recorded with a luminescent image analyzer (LAS-4000; GE Healthcare).

### Chlorophyll fluorescence and P700 analyses

Chlorophyll fluorescence and P700^+^ absorption changes were measured using a Dual-PAM-100 system equipped with a P700 dual-wavelength emitter (830/870 nm; Walz, Effeltrich, Germany). Plants were acclimated to room light for 20 min prior to measurement, and detached leaves were used. Minimal fluorescence in the dark (*F*_o_) was measured with weak measuring light (620 nm, 0.05 to 0.1 µmol photons m^−2^ s^−1^). A saturating pulse (SP; 300 ms, 20,000 µmol photons m^−2^ s^−1^) was applied to determine maximal fluorescence in the dark (*F*_m_) and during actinic illumination ($F_{m}^{\prime }$). Steady-state fluorescence (*F*_s_) was recorded immediately before SP application under actinic light (AL; 635 nm). PSII quantum yield [Y(II)] and NPQ were calculated as ($F_{m}^{\prime }-F_{s}$)/$F_{m}^{\prime }$ and ($F_{m}-F_{m}^{\prime }$)/$F_{m}^{\prime }$, respectively.

Redox changes of P700 were monitored by differential absorbance at 830 and 875 nm. The maximal P700^+^ level in the dark (*P*_m_) was determined by applying an SP under far-red light (720 nm), whereas the maximal P700^+^ level in the light ($P_{m}^{\prime }$) was determined by applying an SP under AL illumination. The steady-state P700^+^ level (*P*) was recorded immediately before SP application. PSI quantum yield [Y(I)] was calculated as (*P*_m_ − *P*)/*P*_m_. Acceptor-side [Y(NA)] and donor-side [Y(ND)] limitations of PSI were calculated as ($P_{m}-P_{m}^{\prime }$)/*P*_m_ and *P*/*P*_m_, respectively. Because P700 exists in 3 mutually exclusive states, Y(I) + Y(NA) + Y(ND) = 1 (Klughammer and Schreiber 1994).

### Measurements of P700 reduction kinetics

Transient redox changes of P700 were measured flash-spectrophotometrically as described previously (Jahns et al. 2002). Thylakoids equivalent to 10 µg chlorophyll mL^−1^ were suspended in a medium containing 5 mm MgCl_2_, 10 mm NaCl, and 0.3 m sorbitol. Buffer systems (5 mm) were used as follows: Tricine/NaOH (pH 7.0 to 8.0), MOPS/NaOH (pH 6.0 to 7.0), and MES/NaOH (pH 5.0 to 6.0). Potassium ferricyanide [K_3_Fe(CN)_6_] was added at a final concentration of 200 µM as an electron acceptor. P700 oxidation was monitored using a Dual-PAM-100 system by applying a single turnover flash (5 µs, 20,000 µmol photons m^−2^ s^−1^) under dark conditions. Forty flashes were applied at 2 s intervals (technical replicates). Signals were recorded with a time resolution of 10 µs, and 20 data points (200 µs) were averaged for normalization.

### In vivo measurements of the ECS signal

The ECS signal was monitored as an absorption change at 515 to 550 nm using a Dual-PAM 100 equipped with a P515/535 module (Walz), as described previously (Yamamoto and Shikanai 2020). The ECS signal was detected after 3 min illumination at different AL intensities (106, 200, 343, and 916 µmol photons m^−2^ s^−1^) using the same leaf, and then, to record ECSt, the AL was turned off for 1 min. *g*_H_^+^ was estimated by fitting the first 300 ms of the decay curve with a first-order exponential decay kinetic as the inverse of the decay time constant (Avenson et al. 2005).

## Supplementary Material

kiag391_Supplementary_Data

## Data Availability

All the information related to this manuscript is included in the Supplementary Materials.
