# Supplementary material for: Regulatory plasticity balances photosynthetic electron flow with enhanced pH-dependent cytochrome b6f control in Arabidopsis
Source: Plant Physiol. 2026 Jun 19;201(3):kiag391. doi: 10.1093/plphys/kiag391 (PMC13421779; doi:10.1093/plphys/kiag391)
Supplement: kiag391_Supplementary_Data [file kiag391_supplementary_data.zip › SI Figs_6410.pdf]

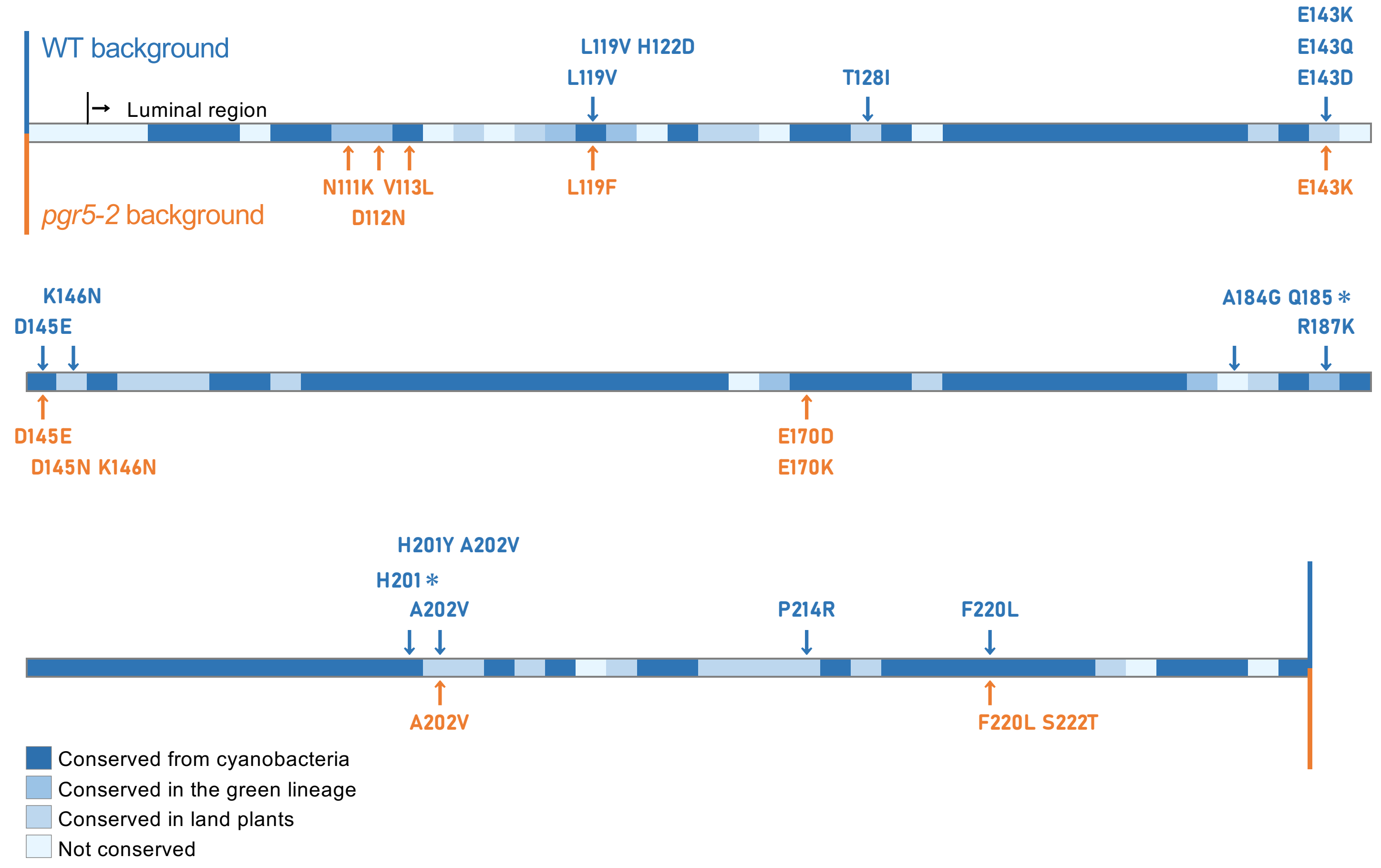

Supplemental Figure S1. Positions of mutations in 26 newly identified *pgr1* alleles. Bars represent the luminal region of the Rieske protein. Shades of blue indicate the degree of amino acid conservation, with darker blue indicating higher conservation. Mutations identified in the wild-type (WT) and *pgr5-2* backgrounds are shown above and below the bars, respectively. Amino acid numbers correspond to the full-length Rieske protein of Arabidopsis, including the plastid-targeting sequence. Asterisks denote stop codons.

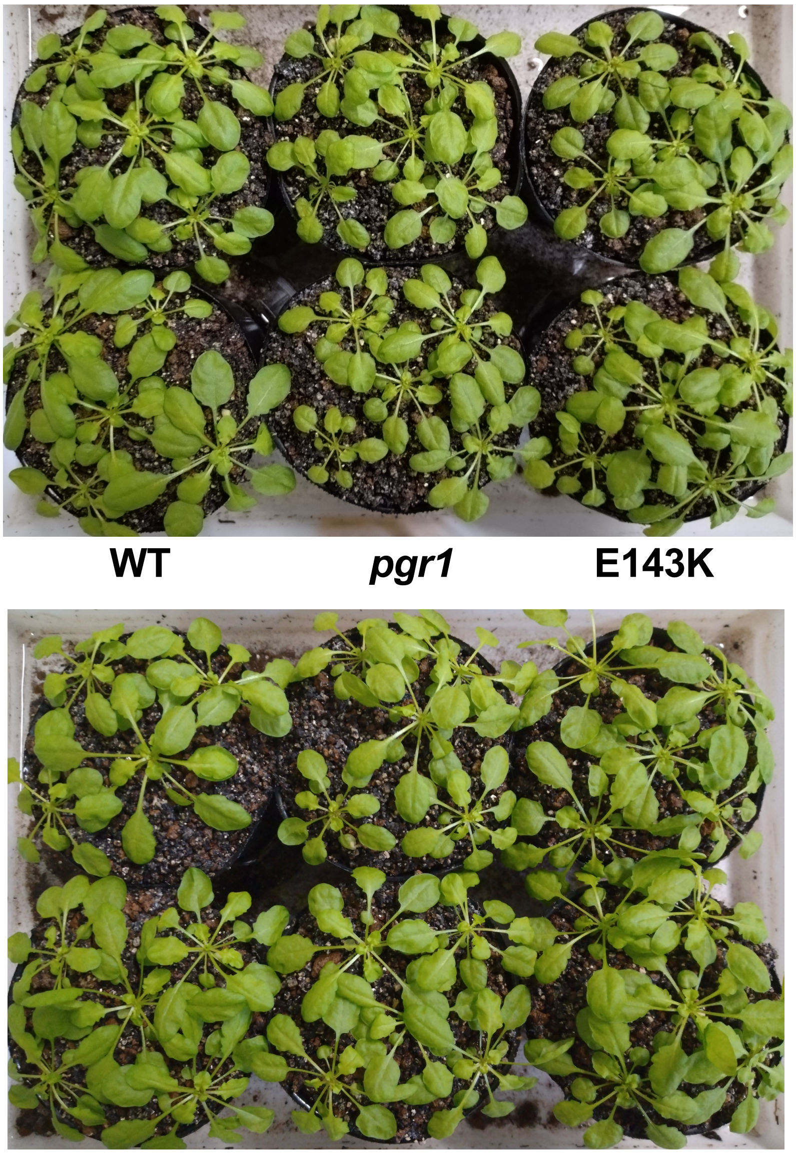

Supplemental Figure S2. Normal growth of the mutants used in this study. E143K mutant plants in the wild-type (WT) and *pgr5-2* backgrounds were grown under short-day conditions (8-h photoperiod at 70 µmol photons m^{-2} s^{-1}) for six weeks. *pgr1*and *pgr1 pgr5-2* plants were grown under the same conditions together with control plants (WT and *pgr5-2*).

a

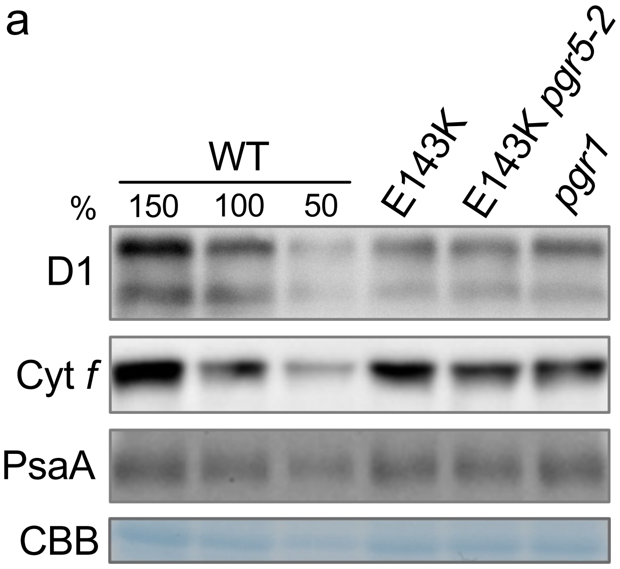

b

	WT 150%	WT 100%	WT 50%	E143K	E143K *pgr5-2*	*pgr1*
Cyt *f* #1	141.3	112.4	46.2	110.4	96.2	102.8
Cyt *f* #2	151.4	96.8	51.7	109.9	106.9	NA
mean	146.35	104.6	48.95	110.15	101.55	102.8
PsaA #1	150.5	98.7	50.6	114.8	97.9	115.2
PsaA #2	146.4	105.9	47.5	112.6	87.1	123.9
mean	148.45	102.3	49.05	113.7	92.5	119.55
D1 #1	152.8	110.8	60.1	82.6	89.8	95.4
D1 #2	145.6	107.1	47.2	73.7	52.4	NA
mean	149.2	108.95	53.65	78.15	71.1	95.4

Supplemental Figure S3. Immunoblot analysis of thylakoid membrane proteins. a) Thylakoid membrane proteins were separated by SDS-PAGE and probed with antibodies against D1 (PSII), cytochrome *f* (Cyt b_6f), and PsaA (PSI) (#1). As a loading control, gels were stained with Coomassie Brilliant Blue (CBB). Each lane contained protein corresponding to 0.5 or 1 µg of chlorophyll. b) Quantitative analysis of immunoblot signals, including a biological replicate (#2). Values were estimated from a calibration curve generated from WT samples loaded at 50%, 100%, and 150% levels. In the #2 experiment, the *pgr1* mutant was not analyzed with two antibodies (NA).

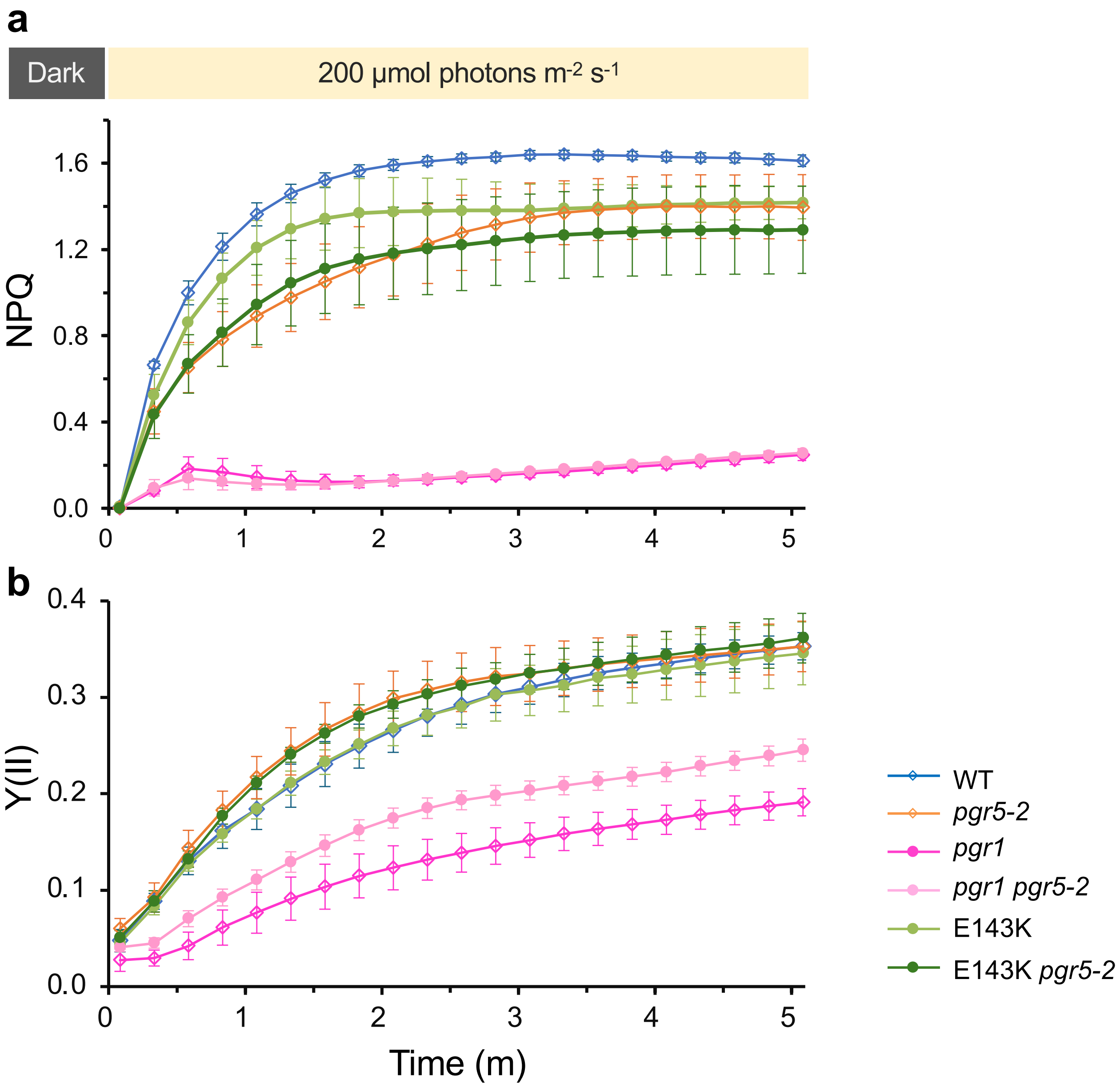

Supplemental Figure S4. Induction of electron transport. Photosynthesis was induced by non-saturating actinic light (200 μmol photons m^{-2} s^{-1}). The parameters Y(II) a) and NPQ b) were measured using a Dual-PAM-100 system in the same analysis shown in Figure 3. Detached leaves from dark-adapted plants were exposed to actinic light. Statistical analyses are summarized in Supplemental Table S3.

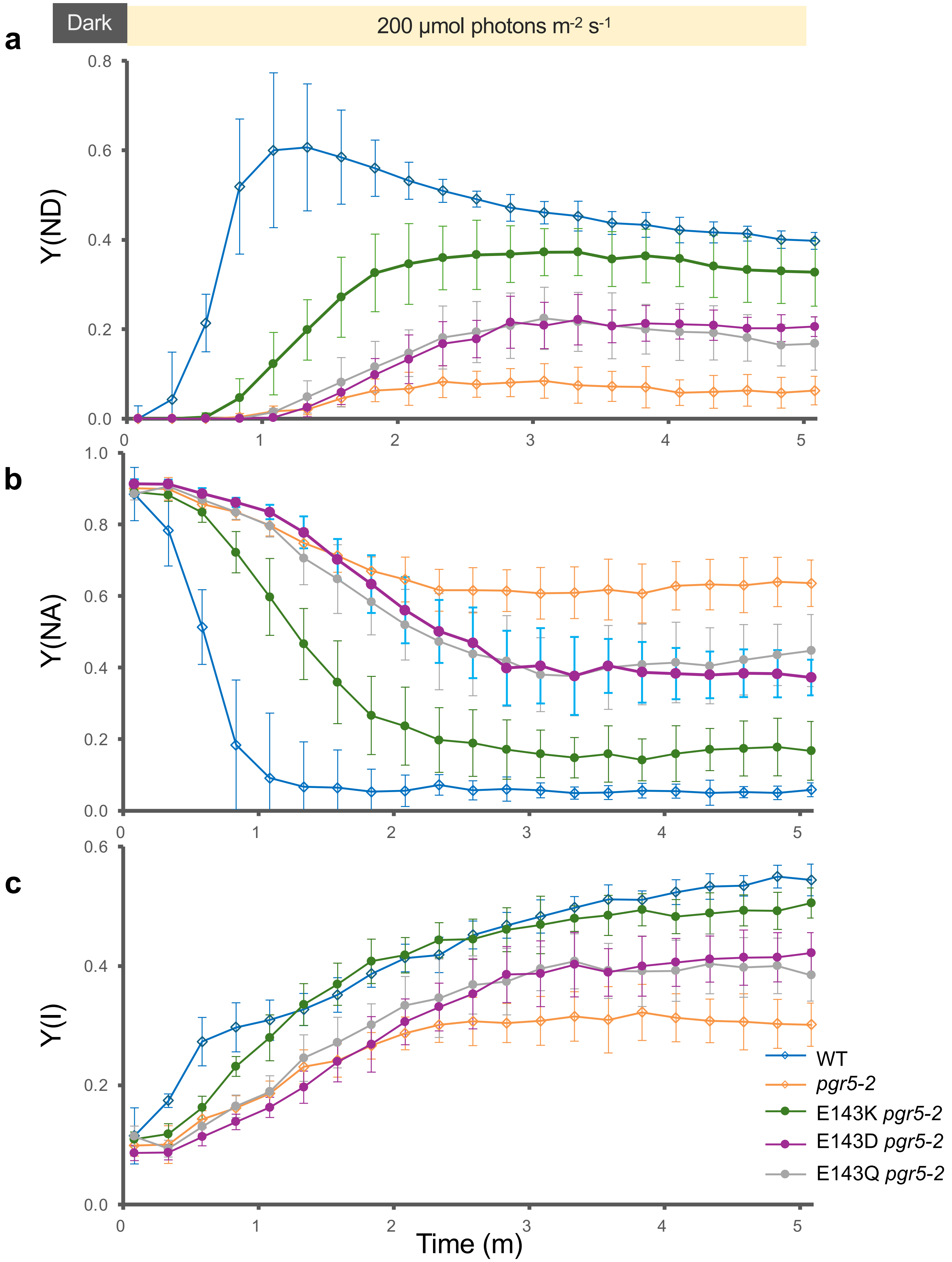

Supplemental Figure S5. Induction of electron transport in the E143D *pgr5-2* and E143Q *pgr5-2* alleles. Photosynthesis was induced under non-saturating actinic light (200 µmol photons m^{-2} s^{-1}). The parameters Y(ND) a), Y(NA) b), and Y(I) c) were measured with a Dual-PAM-100 system. Detached leaves from dark-adapted plants were exposed to actinic light. As controls, the same data as in Figure 3 are shown for WT, *pgr5-2*, and E143K *pgr5-2*. Statistical analyses were conducted using the Tukey-Kramer test and are summarized in Supplemental Table S4.

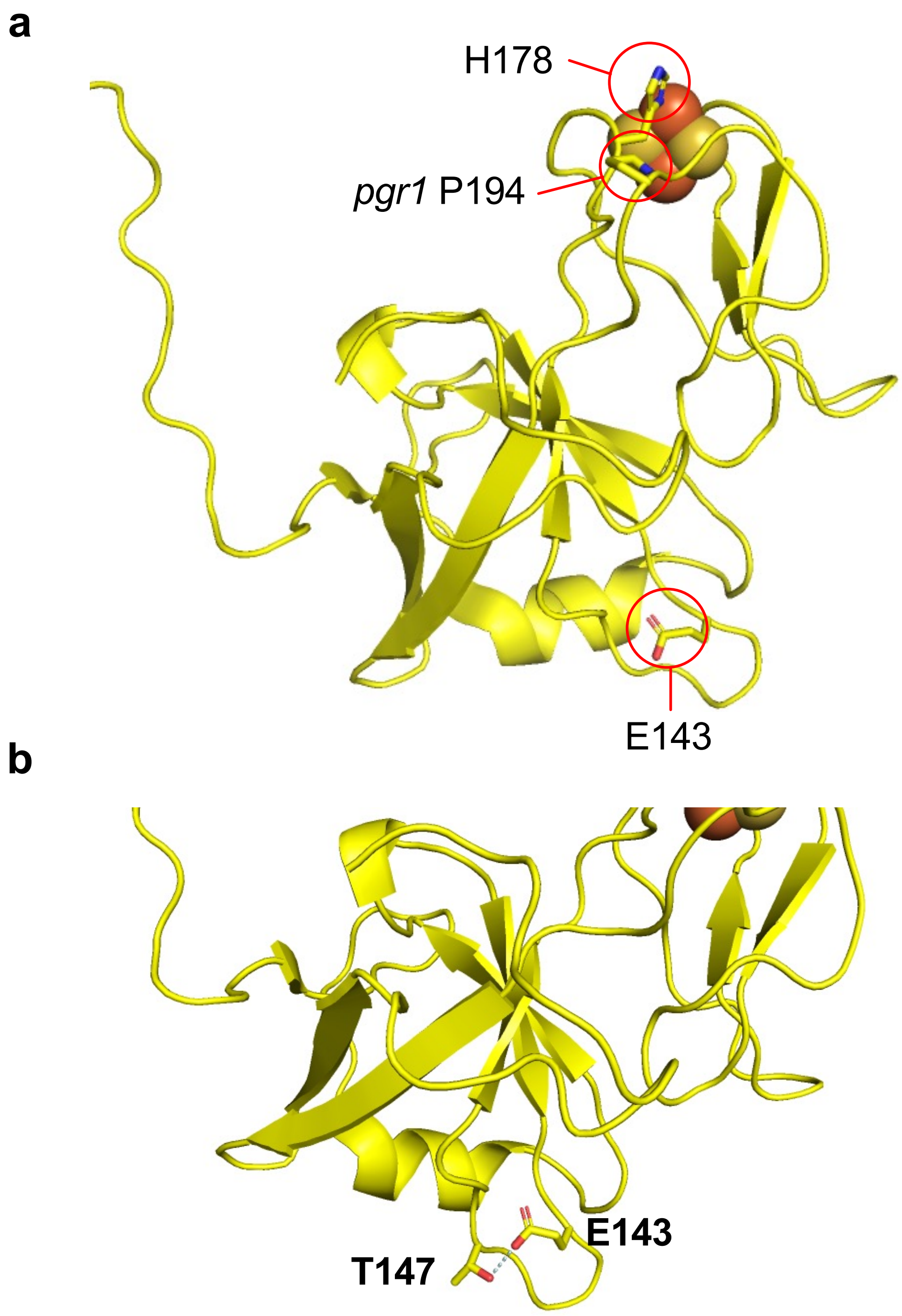

Supplemental Figure S6. Structure of the luminal region of the Rieske subunit. The spinach structure (PDB ID: 6RQF) was obtained from the Protein Data Bank Japan (PDBj). a) The positions of E143 and the *pgr1* mutation (P194) are indicated, together with H178, which may sense luminal acidification. b) E143 is predicted to form a hydrogen bond with T147.

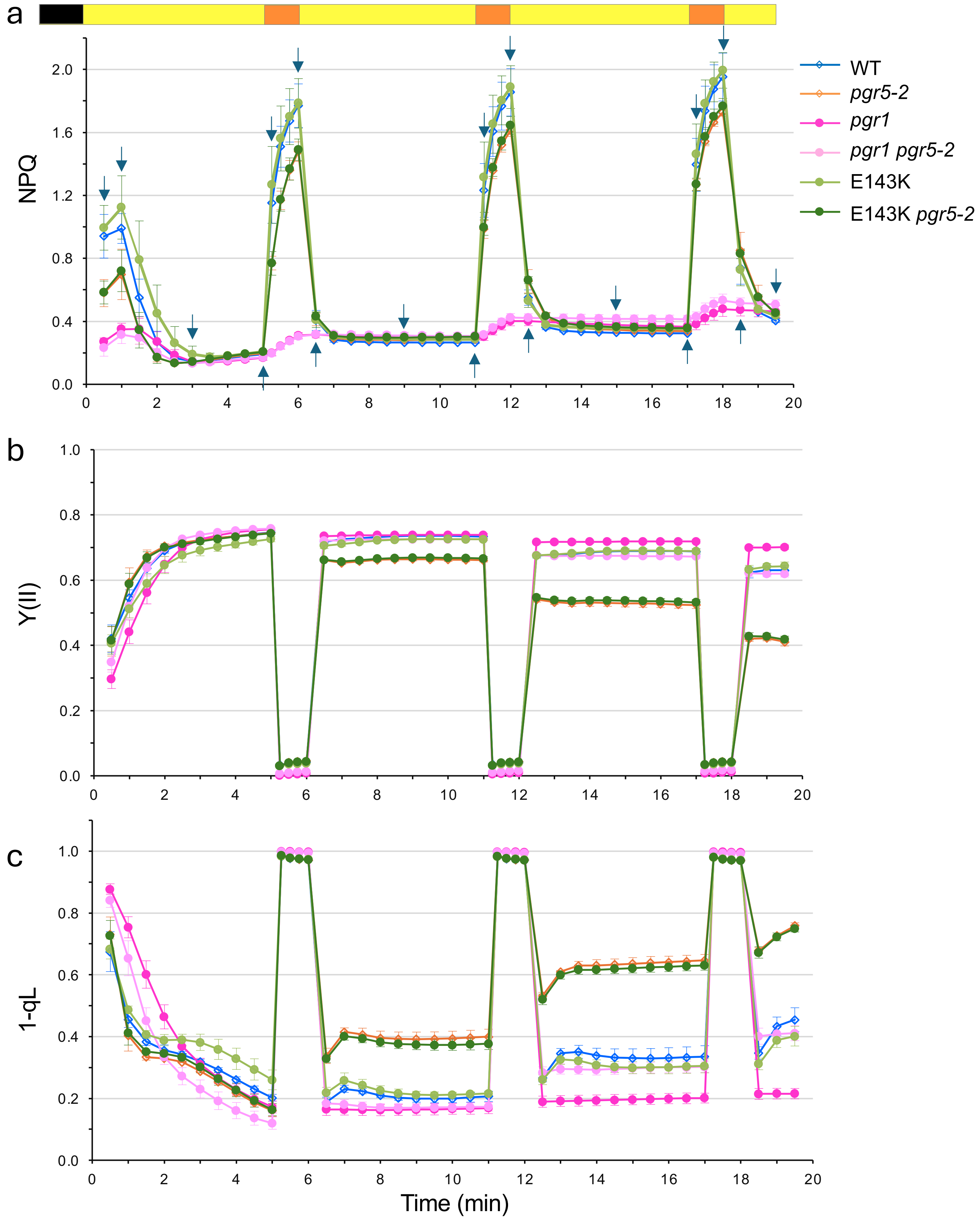

Supplemental Figure S7. Chlorophyll fluorescence parameters under fluctuating light. The fluctuating light condition consists of 5-min LL (yellow bars) of 50 μmol photons m $^{-2}$ s $^{-1}$ and 1-min HL (orange bars) of 1,510 μmol photons m $^{-2}$ s $^{-1}$. A black bar represents the adaptation to the dark. NPQ(a), Y(II) (b), and 1-qL (c) were analyzed in detached leaves from each genotype indicated. Analysis performed simultaneously with the P700 analyses (Fig. 5). Data represent mean ± SD (*n* = 5, biological replicates). Statistical analyses were conducted using the Tukey-Kramer test and are summarized in Supplemental Table S6. Vertical arrows in a indicate time points used for the statistical analyses.

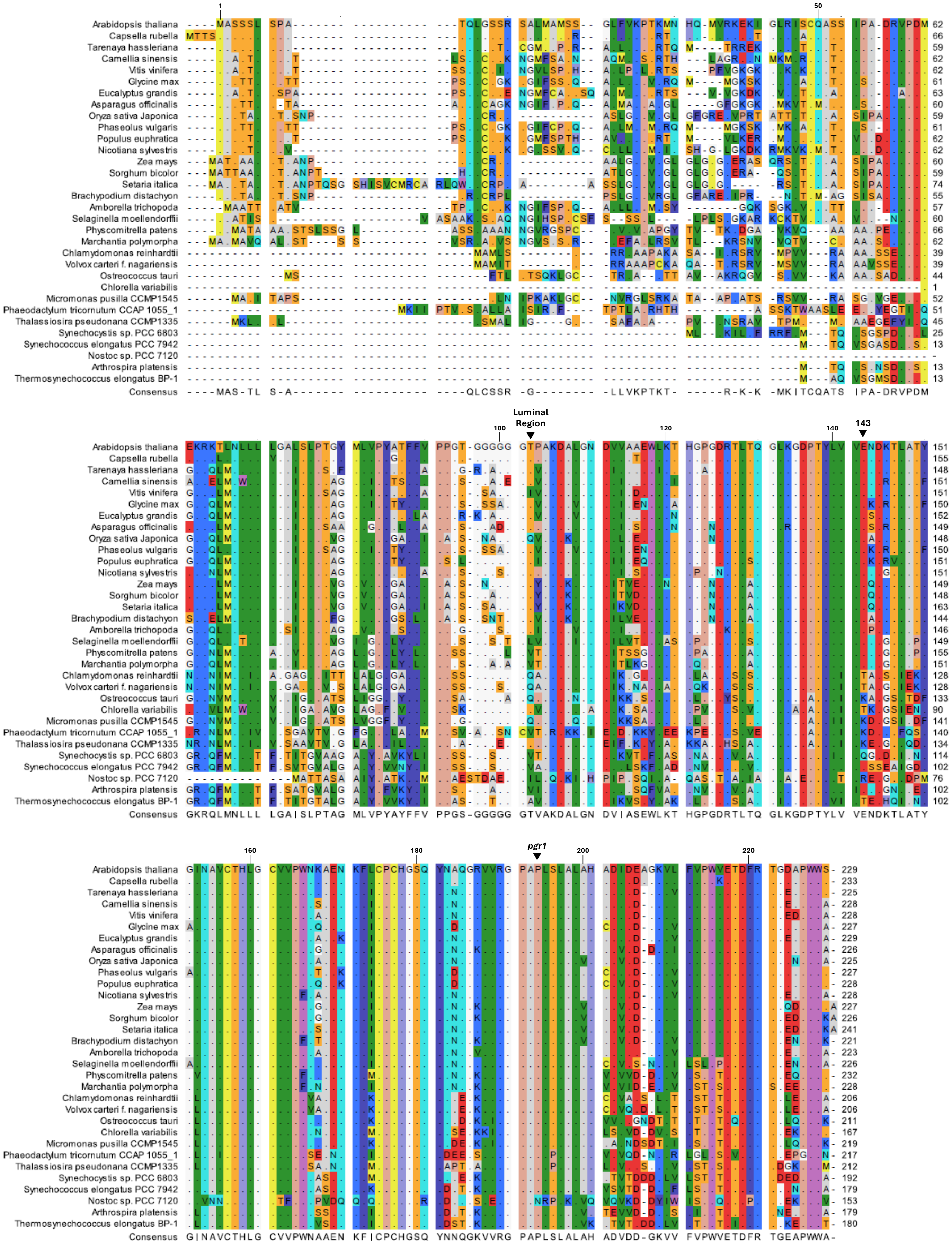

Supplemental Figure S8. Alignment of amino acid sequences of Rieske proteins from cyanobacteria to angiosperms. Numbers above the sequences correspond to the Arabidopsis Rieske protein shown at the top. The positions of the E143K and *pgr1* mutations are indicated. The luminal region begins at Thr103.
